# BacCapSeq: a Platform for Diagnosis and Characterization of Bacterial Infections

**DOI:** 10.1128/mBio.02007-18

**Published:** 2018-10-23

**Authors:** Orchid M. Allicock, Cheng Guo, Anne-Catrin Uhlemann, Susan Whittier, Lokendra V. Chauhan, Joel Garcia, Adam Price, Stephen S. Morse, Nischay Mishra, Thomas Briese, W. Ian Lipkin

**Affiliations:** a Center for Infection and Immunity, Mailman School of Public Health, Columbia University, New York, New York, USA; b Department of Medicine, Infectious Diseases, Vagelos College of Physicians and Surgeons, Columbia University, New York, New York, USA; c Department of Pathology and Cell Biology, Vagelos College of Physicians and Surgeons, Columbia University, New York, New York, USA; d Department of Epidemiology, Mailman School of Public Health, Columbia University, New York, New York, USA; e Department of Neurology, Mailman School of Public Health and Vagelos College of Physicians and Surgeons, Columbia University, New York, New York, USA; University of Texas at Austin; University of Maryland School of Medicine; New York University

**Keywords:** antibiotic resistance, bacterial identification, diagnostics, sequencing

## Abstract

We report a platform that increases the sensitivity of high-throughput sequencing for detection and characterization of bacteria, virulence determinants, and antimicrobial resistance (AMR) genes. The system uses a probe set comprised of 4.2 million oligonucleotides based on the Pathosystems Resource Integration Center (PATRIC) database, the Comprehensive Antibiotic Resistance Database (CARD), and the Virulence Factor Database (VFDB), representing 307 bacterial species that include all known human-pathogenic species, known antimicrobial resistance genes, and known virulence factors, respectively. The use of bacterial capture sequencing (BacCapSeq) resulted in an up to 1,000-fold increase in bacterial reads from blood samples and lowered the limit of detection by 1 to 2 orders of magnitude compared to conventional unbiased high-throughput sequencing, down to a level comparable to that of agent-specific real-time PCR with as few as 5 million total reads generated per sample. It detected not only the presence of AMR genes but also biomarkers for AMR that included both constitutive and differentially expressed transcripts.

## Abstract (precis)

BacCapSeq is a method for differential diagnosis of bacterial infections and defining antimicrobial sensitivity profiles that has the potential to reduce morbidity and mortality, health care costs, and the inappropriate use of antibiotics that contributes to the development of antimicrobial resistance.

## INTRODUCTION

More than 1.5 million individuals annually develop sepsis in the United States alone; approximately 250,000 have fatal outcomes (1). Current diagnosis of sepsis is culture based and may require 48 to 72 h (2, 3). An additional challenge is that some pathogenic bacteria are fastidious, such as Legionella or Bartonella species, and difficult to cultivate. Multiplex PCR systems have been established for differential diagnosis of infection detecting up to 19 pathogenic bacteria (4). However, no platform currently permits rapid and simultaneous insights into phylogeny, pathogenicity markers, and antimicrobial resistance (AMR) needed to enable the early and precise antibiotic treatment that could reduce morbidity, mortality, and economic burden.

The strategy of targeted sequence capture has been employed in the assessment of the human exome and individual targeted genes (5–7) and for viral discovery, surveillance, and diagnostics (8, 9). Here, we describe a bacterial capture sequence system, BacCapSeq, modeled on VirCapSeq-VERT, an analogous system developed to detect all known viruses infecting vertebrates (10).

## RESULTS

### Probe design strategy.

**FIG 1:**
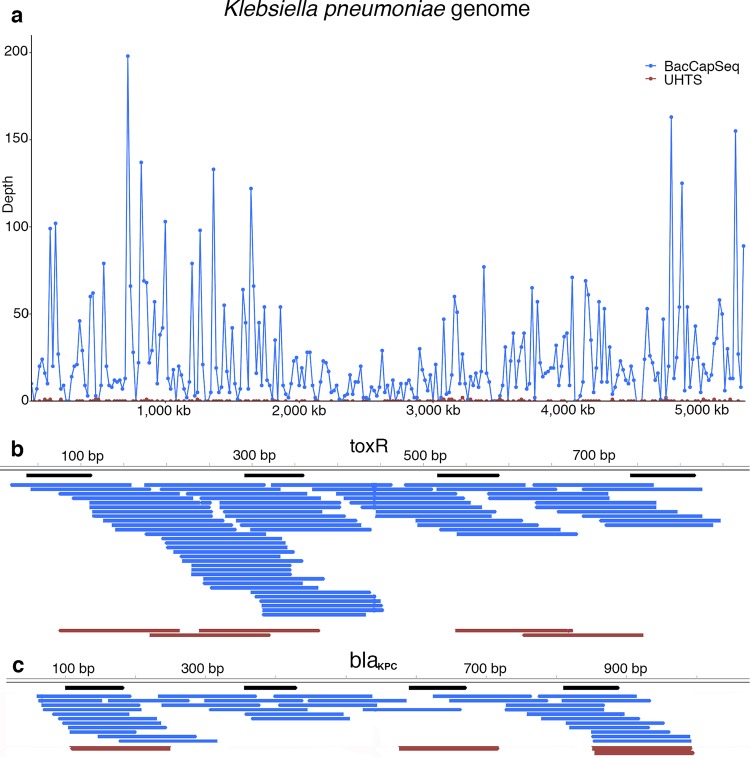
BacCapSeq yields more reads and higher genome coverage than unbiased high-throughput sequencing. (A) Graphic representation of read depth obtained with BacCapSeq or UHTS across the K. pneumoniae genome. (B) Representative BacCapSeq results for the *toxR* virulence gene obtained from whole-blood nucleic acid spiked with 40,000 copies/ml of V. cholerae DNA and (C) the *bla*_KPC_ AMR gene obtained from whole blood spiked with 40,000 live K. pneumoniae cells/ml. Probes are shown in black. BacCapSeq reads are shown in blue. UHTS reads are shown in brown.

We assembled a probe set comprised of 4.2 million oligonucleotides based on the Pathosystems Resource Integration Center (PATRIC) database (11), representing 307 bacterial species that include all known human-pathogenic species. The probe set also represents all known antimicrobial resistance genes and virulence factors based on sequences in the Comprehensive Antibiotic Resistance Database (CARD) (12) and Virulence Factor Database (VFDB) (13, 14). Probes were selected along the coding sequences of the 307 targeted bacteria (see Table S1 in the supplemental material) with an average length of 75 nucleotides (nt) to maintain a probe melting temperature (*T_m_*) with a mean of 79°C. The average interval between probes along annotated protein coding sequences targeted for capture was 121 nt (see Materials and Methods for further details). The probes capture fragments that include sequences contiguous to their targets; thus, we recovered near complete protein coding sequence. An example with Klebsiella pneumoniae is shown in Fig. 1a. Probes based on the CARD and VFDB databases ensured coverage of AMR genes and virulence factors, as illustrated by detection of the *toxR* virulence factor regulator in Vibrio cholerae (Fig. 1b) and *bla*_KPC_ AMR gene in K. pneumoniae (Fig. 1c).

10.1128/mBio.02007-18.1 TABLE S1 Bacteria targeted in BacCapSeq. Download Table S1, XLSX file, 0.1 MB. Copyright © 2018 Allicock et al. 2018 Allicock et al. This content is distributed under the terms of the Creative Commons Attribution 4.0 International license.

### BacCapSeq performance using whole-blood nucleic acid spiked with bacterial nucleic acid.

The efficiency of the bacterial capture sequence (BacCapSeq) system versus conventional unbiased high-throughput sequencing (UHTS) was assessed in side-by-side comparisons of data obtained with 5 million reads per sample. We began with extracts of whole blood spiked with DNA from Bordetella pertussis, Escherichia coli, Neisseria meningitidis, Salmonella enterica serovar Typhi, Streptococcus agalactiae, Streptococcus pneumoniae, V. cholerae, and Campylobacter jejuni at concentrations ranging from 40 to 40,000 copies/ml. BacCapSeq yielded up to 100-fold more reads and higher genome coverage for all bacterial targets tested compared to UHTS (Table 1). The enhanced performance of BacCapSeq was particularly pronounced at lower copy numbers.

**TABLE 1:** BacCapSeq yields higher read counts and genome coverage than UHTS in whole-blood extracts spiked with bacterial DNA

Species	Genome length (nt)	Coding region (%)	Load (copies/ml)	Bacterial read count^*a*^		Fold increase	Genome coverage (%)	
				BacCapSeq	UHTS		BacCapSeq	UHTS
Bordetella pertussis	4,386,396	89	40,000	329,926	203,563	2	100	99
			4,000	295,830	19,362	15	98	93
			400	155,109	2,189	71	73	29
			40	8,596	191	45	9	3
Escherichia coli	4,965,553	88	40,000	281,925	77,793	4	82	81
			4,000	253,423	7,558	34	81	60
			400	132,168	848	156	64	11
			40	8,614	70	123	8	1
Neisseria meningitidis	2,272,360	86	40,000	228,937	72,532	3	93	93
			4,000	206,096	6,995	29	91	82
			400	109,446	824	133	79	22
			40	6,609	68	97	13	2
Salmonella enterica serovar Typhi	4,791,961	88	40,000	25,155	8,620	3	94	63
			4,000	22,726	841	27	68	12
			400	12,009	102	118	16	1
			40	796	10	80	1	0
Streptococcus agalactiae	2,198,785	89	40,000	8,467	4,701	2	85	67
			4,000	7,905	473	17	63	15
			400	4,206	58	73	13	2
			40	298	4	75	1	0
Streptococcus pneumoniae	2,038,615	86	40,000	8,419	2,920	3	91	56
			4,000	7,795	280	28	66	10
			400	4,124	30	137	14	1
			40	275	2	138	1	0
Vibrio cholerae	6,048,147	87	40,000	11,291	5,381	2	97	64
			4,000	10,124	530	19	66	12
			400	5,127	61	84	12	1
			40	315	6	53	1	0
Campylobacter jejuni	1,641,481	94	40,000	5,904	4,195	1	89	73
			4,000	5,460	415	13	63	17
			400	3,223	52	62	14	2
			40	235	3	78	1	0

a Bacterial reads per 1 million reads are shown without applying a cutoff threshold.

### BacCapSeq performance using whole blood spiked with bacterial cells.

**FIG 2:**
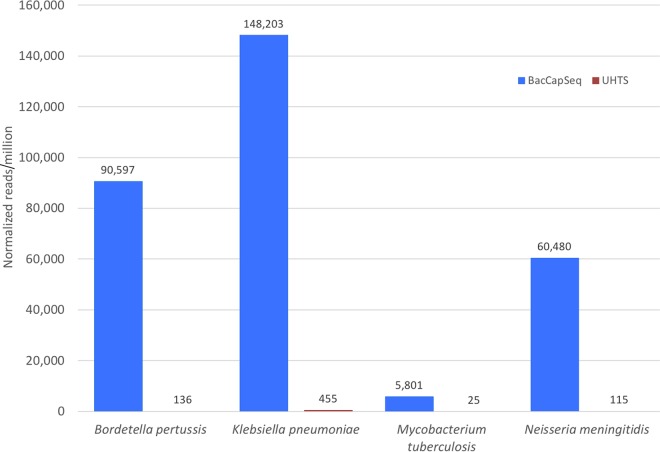
Mapped bacterial reads in blood spiked with bacterial cells. Mapped bacterial reads were normalized to 1 million quality- and host-filtered reads obtained by BacCapSeq (blue) or UHTS (brown). The data shown represent 40,000 cells/ml. No cutoff threshold was applied.

We tested performance with whole blood spiked with K. pneumoniae, B. pertussis, N. meningitidis, S. pneumoniae, and Mycobacterium tuberculosis cells. Nucleic acid was extracted from the spiked sample and processed for BacCapSeq or UHTS. Here too, BacCapSeq yielded more reads and higher genome coverage than UHTS (Table 2 and Fig. 2).

**TABLE 2:** BacCapSeq yields higher read counts and genome coverage than UHTS in whole blood spiked with bacterial cells

Species	Genome length (nt)	Coding region (%)	Load (copies/ml)	Bacterial read count^*a*^		Fold increase	Genome coverage (%)	
				BacCapSeq	UHTS		BacCapSeq	UHTS
Bordetella pertussis	4,386,396	89	40,000	90,597	136	694	82	9
			4,000	14,858	16	979	39	5
			400	1,622	2	725	13	1
			40	269	1	508	8	0
Klebsiella pneumoniae	5,333,942	89	40,000	148,203	455	339	92	6
			4,000	16,929	40	442	58	1
			400	2,771	5	551	18	0
			40	522	0	NA^*b*^	5	0
Mycobacterium tuberculosis	4,411,532	91	40,000	5,801	25	243	46	0
			4,000	845	3	287	9	0
			400	14	0	NA	0	0
			40	6	0	NA	0	0
Neisseria meningitidis	2,272,360	86	40,000	60,480	115	546	90	6
			4,000	6,894	8	908	57	0
			400	1,454	1	1,561	23	0
			40	151	0	NA	6	0
Streptococcus pneumoniae	2,038,615	86	40,000	3,070	6	506	43	0
			4,000	588	1	948	13	0
			400	35	0	NA	1	0
			40	4	0	NA	0	0

a Bacterial reads per 1 million reads are shown without applying a cutoff threshold.

b NA, not applicable because fold increase was not calculated for results with <1 read.

### BacCapSeq performance using cultured blood samples.

We tested the utility of BacCapSeq in analysis of blood culture samples obtained from the Clinical Microbiology Laboratory at New York-Presbyterian Hospital/Columbia University Medical Center. Patient blood was collected into conventional BacTec blood culture flasks and incubated until flagged as growth positive by the BD BacTec automated blood culture system (Becton Dickinson). Using BacCapSeq, we recovered near full genome sequence and identified antimicrobial resistance genes that matched standard clinical microbiology laboratory antimicrobial sensitivity testing (AST) profiles (Table 3; see Table S2 in the supplemental material).

**TABLE 3:** Detection of pathogenic bacteria and antimicrobial resistance genes in cultured blood samples

Sample	No. of raw reads	Total no. of mapped reads	Bacterium identified	Genome coverage (%)	AST profile^*a*^	Significant AMR gene(s) detected
1	2,833,697	2,709,612	Pseudomonas aeruginosa	87	TET (R), MERO (I)	*mexA* to -*N*, -*P*, -*Q*, -*S*, -*V*, and -*W* combined with *oprM*
2	8,322,222	7,126,518	Escherichia coli	81	AMP (I), CEF (I)	TEMs (115, 4, 80, 6, 153, 143, 79), combined with numerous efflux pump antiporters (including most prominently *acrF*, *cpxR*, or H-NS)
3	5,768,129	5,096,360	Morganella morganii	90	AMP (R), CEPH (R), AZT (I)	Numerous DHA complex β-lactamases (DHA-20, -17, -21, -1, -19), combined with efflux pump antiporters *acrB* and *smeB*; *cpxR*, related to aztreonam resistance
4	5,749,637	4,774,301	Haemophilus influenzae	92	NA	*hmrM*

a Antimicrobial sensitivity test (AST) profile: AMP, ampicillin; AZT, aztreonam; CEF, cefoxitin; CEPH, cefazolin/ceftazidime/ceftriaxone; MERO, meropenem; TET, tetracycline. R, resistant; I, intermediate rating; NA, not applicable.

10.1128/mBio.02007-18.2 TABLE S2 Antimicrobial resistance genes detected in cultured blood samples. Download Table S2, XLSX file, 0.1 MB. Copyright © 2018 Allicock et al. 2018 Allicock et al. This content is distributed under the terms of the Creative Commons Attribution 4.0 International license.

### BacCapSeq performance with human blood samples.

**FIG 3:**
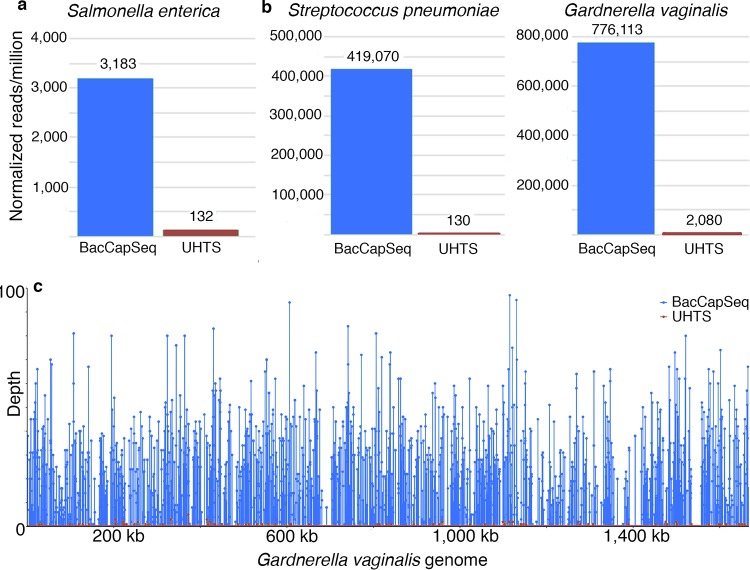
Identification of bacteria in two immunosuppressed patients with HIV/AIDS and unexplained sepsis. (A) Infection with Salmonella enterica; (B) coinfection with Streptococcus pneumoniae and Gardnerella vaginalis; (C) genomic coverage of Gardnerella vaginalis.

Blood samples from two immunosuppressed individuals with HIV/AIDS and sepsis of unknown cause were extracted and processed for BacCapSeq and UHTS analysis in parallel. A causative agent was identified by either method; however, BacCapSeq yielded higher numbers of relevant reads and better genome coverage (Fig. 3). Salmonella enterica was detected in one patient. The other patient had evidence of coinfection with both S. pneumoniae and Gardnerella vaginalis.

### BacCapSeq-facilitated discovery of potential AMR biomarkers.

**FIG 4:**
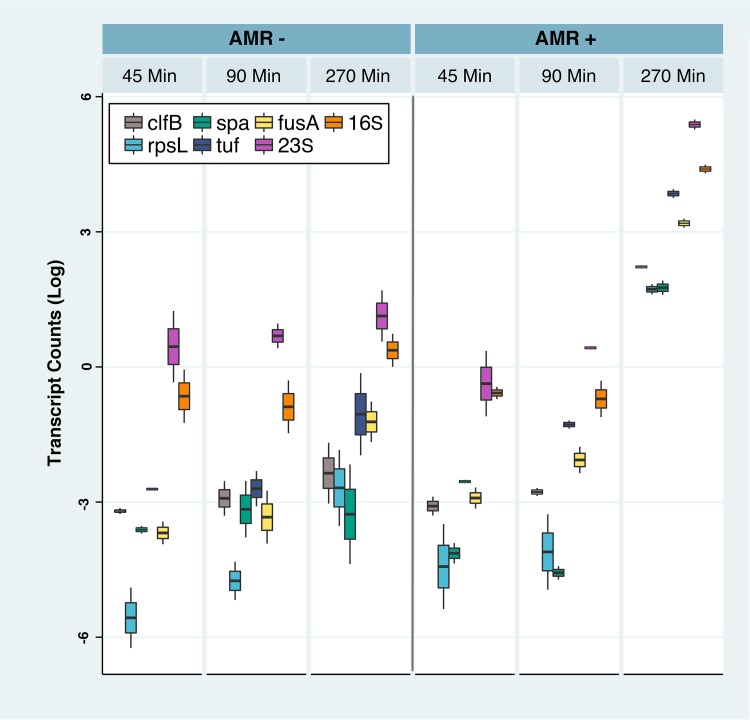
Levels of seven transcripts in Staphylococcus aureus sensitive (AMR−) or resistant (AMR+) to ampicillin after culture for 45, 90, and 270 min in the presence of ampicillin. Box plots represent the log of normalized transcript counts for each gene. Only results obtained with BacCapSeq are shown because no transcripts were detected in the presence of ampicillin with UHTS until later time points.

The current probe set specifically captures all AMR genes present in the CARD database. Demonstrating the presence of an AMR gene is not equivalent to finding evidence for its functional expression. To address this challenge, we have begun to use BacCapSeq to pursue potential biomarkers in bacteria exposed to antibiotics. We cultured ampicillin-sensitive and -resistant strains of Staphylococcus aureus at an inoculum of 1,000 CFU/ml in the presence or absence of the antibiotic for 45, 90, and 270 min. We then extracted RNA for BacCapSeq and UHTS for transcriptomic analysis to find biomarkers that differentiated ampicillin-sensitive and ampicillin-resistant S. aureus strains exposed to ampicillin. As illustrated in Fig. 4, BacCapSeq enabled discovery of transcripts that were differentially expressed between 90 min and 270 min of antibiotic exposure. These represented constitutive genes that reflect bacterial replication, including classical strain- and species-specific markers such as 16S and 23S rRNA, elongation factors Tu (*tuf*) and G (*fusA*), protein A (*spa*), clumping factor B (*clfB*), or 30S ribosomal protein S12 (*rpsL*).

## DISCUSSION

In the pre-antibiotic era, puerperal sepsis was a common cause of maternal mortality. Up to 30% of children did not survive their first year of life, and community-acquired pneumonia and meningitis resulted in 30% and 70% mortality, respectively (15). The advent of bacterial diagnostics and antibiotics has not only reduced the burden of naturally occurring infectious diseases, but has also enhanced our quality of life by enabling innovations in clinical medicine such as organ transplantation, joint replacement, and other invasive surgical procedures, immunosuppressive chemotherapy, and burn management (16). These advances are threatened by the emergence of AMR. In 2013, the collaborative World Economic Forum estimated 100,000 annual AMR-related deaths in the United States alone due to hospital-acquired infections (17). The global impact of AMR is estimated at 700,000 deaths annually, with the highest burden in the developing world. The financial implications are also dire. The CDC estimates the direct annual impact of antibiotic resistance in the United States is 20 to 35 billion U.S. dollars (USD), with an additional 35 billion USD in lost productivity (18). Absent an effective response to limit further growth in AMR, the challenge will continue to increase. The World Bank issued a report in March 2017 citing the impact of AMR on the gross domestic product (GDP) by 2030 will be between 1.1 trillion and 3.4 trillion USD (19).

Early, accurate differential diagnosis of bacterial infections is critical to reducing morbidity, mortality, and health care costs. It can also enhance antibiotic stewardship by reducing the inappropriate use of antibiotics. Multiplex PCR methods in common use for differential diagnosis of bacterial infections can identify potential pathogens but do not provide insights into the presence or expression of AMR genes. Furthermore, they do not include bacteria only rarely associated with significant disease, such as G. vaginalis, implicated here in unexplained sepsis in an individual with HIV/AIDS. Culture-based methods require 2 to several days to identify pathogens and even longer to provide antibiotic susceptibility profiles (20, 21). Accordingly, physicians typically administer broad-spectrum antibiotics pending acquisition of more specific information (22).

We have not subjected BacCapSeq to rigorous validation using methods for determining limits of detection and reproducibility required for approval of a diagnostic assay in clinical microbiology. Nonetheless, results obtained with blood samples spiked with known concentrations of bacterial DNA (Table 1 and Fig. 1) or bacterial cells (Table 2 and Fig. 2) demonstrated a dose-dependent, consistent enhancement in the number of reads recovered and genome coverage obtained with BacCapSeq versus UHTS. In instances where the bacterial load was as low as 40 cells per ml, UHTS detected no sequences of M. tuberculosis, K. pneumoniae, N. meningitidis, or S. pneumoniae and only one read for B. pertussis. In each of these instances, BacCapSeq detected multiple reads (M. tuberculosis, 6; K. pneumoniae, 522; N. meningitidis, 151; S. pneumoniae, 4; B. pertussis, 269) (Table 2). This advantage was also observed in analysis of blood from patients with unexplained sepsis (Fig. 3), where reads obtained were higher with BacCapSeq than UHTS for S. enterica (3,183 versus 132), S. pneumoniae (419,070 versus 130), and G. vaginalis (776,113 versus 2,080). These findings suggest that where levels of bacteria in blood are below 40 cells per ml, BacCapSeq has the potential to indicate the presence of a causal pathogen that might be missed by UHTS.

Incubation periods in blood culture systems commonly range from 3 days to 5 days (23–25). Longer intervals may be required for sensitive detection of some pathogenic species of Neisseria, Rickettsia, Mycobacterium, Leptospira, Ehrlichia, Coxiella, Campylobacter, Burkholderia, Brucella, Bordetella, and Bartonella. An additional challenge is that bacterial loads may be low or intermittent. Cockerill et al. (24) and Lee et al. (26) have suggested that 80 ml of blood in four separate collections of at least 20 ml of blood are required for 99% test sensitivity in detecting viable bacteria. We have not performed head-to-head comparisons of either BacCapSeq or UHTS with culture. We also cannot address the issue of intermittent bacteremia but are nonetheless encouraged by the observation that current estimates of BacCapSeq sensitivity (a minimum of 40 copies per ml) correspond favorably to the 80 ml sample volume recommended in culture tests (26). The American Society for Microbiology and the Clinical and Laboratory Standards Institute (CLSI) require false-positivity rates below 3% (27, 28). Protocols for hygiene in diagnostic microbiology will be even more stringent with BacCapSeq than culture because nucleic acids are not eliminated by common disinfectants. To limit potential false-positive calls based on spurious read signals, we propose a conservative threshold cutoff for bona fide signal of 10 reads per million reads. However, the presence of fewer reads specific for the particular strain or isolate should trigger a search for confirmation or refutation using alternative methods, such as specific PCR assays based on findings with BacCapSeq or UHTS.

BacCapSeq is designed to detect all AMR genes in the CARD database. Where these genes are located on bacterial chromosomes, we anticipate that flanking sequences will allow association with specific bacteria within a sample, even when those samples contain more than one bacterial species. Where AMR genes are extrachromosomal, we may not be able to do so. However, BacCapSeq is anticipated to enable the discovery of constitutively expressed and induced transcripts that reflect the presence of functional bacterium-specific AMR elements.

Our objective in building BacCapSeq was to enable efficient and sensitive detection of pathogenic bacteria, virulence factors, and antimicrobial resistance genes. As with VirCapSeq-VERT, the analogous sequence capture platform established for viral diagnostics and surveillance, BacCapSeq is anticipated to allow focused investment of sequencing capacity. This should facilitate multiplexing and decreases in sequencing costs and in the complexity of bioinformatic analysis.

We acknowledge that the system has several constraints. The method is sensitive to as few as 40 to 400 copies/ml. However, BacCapSeq is qualitative, not quantitative. Where measurements of bacterial burden are critical, BacCapSeq findings should be followed by quantitative PCR (qPCR) assays. Such assays would add an additional 3 h to processing time. The system can only detect bacterial sequences that are sufficiently similar to probes for efficient capture. In earlier work, based on probes with similar lengths and probe melting temperature (*T_m_*) properties, we defined a homology threshold for capture of 60% nucleotide identity (10). Accordingly, a novel bacterium that differs from any known human pathogen by more than 40% over the entire length of its coding sequence would not be detected. At present, the time from sample acquisition to results using the Illumina platform is approximately 70 h. This is not a time frame that will have a major impact on clinical diagnostics. However, the probe set is platform agnostic and should be adaptable to nanopore and anticipated more rapid sequencing systems that would have broader utility in clinical microbiology laboratories and resource-challenged environments, such as those found in the developing world. Bacterial typing and AMR biomarker discoveries enabled by BacCapSeq could also be used to establish qPCR assays that produce results within hours of clinical sample receipt.

## MATERIALS AND METHODS

### Bacteria.

The following bacteria were obtained through the NIH Biodefense and Emerging Infections Research Resources Repository, NIAID, NIH: Streptococcus pneumoniae strain SPEC6C, NR-20805; Bordetella pertussis strain H921, NR-42457; Streptococcus agalactiae strain SGBS001, NR-44125; Salmonella enterica subsp. *enterica*, strain Ty2 (serovar Typhi), NR-514; Neisseria meningitidis strain 98008, NR-30536; Klebsiella pneumoniae isolate 1, NR-15410; Escherichia coli strain B171, NR-9296; Vibrio cholerae strain 395, NR-9906; and Campylobacter jejuni strain HB95-29, NR-402. Staphylococcus aureus ATCC 25923 and ATCC 29213 were acquired from the American Type Culture Collection.

### Nucleic acid extraction.

Total nucleic acid from bacterial cells, whole blood spiked with bacteria, or bacterial nucleic acids were extracted using an Allprep mini DNA/RNA kit (Qiagen, Hilden, Germany) and quantitated by NanoDrop One (Wilmington, DE) or Bioanalyzer 2100 (Agilent, Santa Clara, CA). Bacterial nucleic acid and genome equivalents were quantitated by agent-specific quantitative TaqMan real-time PCR.

### Agent-specific quantitative TaqMan real-time PCR and standards.

Primers and probes for quantitative PCR (qPCR) were selected in conserved single-copy genes of the investigated bacterial species with Geneious v10.2.3 (https://www.geneious.com) (Table S3). Standards for quantitation were generated by cloning a fragment of the targeted gene spanning the primers into pGEM-T Easy vector (Promega, Madison, WI). Recombinant plasmid DNA was purified using a Mini Plasmid Prep kit (Qiagen). The linearized plasmid DNA concentration was determined using NanoDrop One, and copy numbers were adjusted by dilution in Tris-HCl (pH 8) with 1 ng/ml salmon sperm DNA.

10.1128/mBio.02007-18.3 TABLE S3 Bacteria and polymerase chain reaction assays used in BacCapSeq validation. Download Table S3, XLSX file, 0.1 MB. Copyright © 2018 Allicock et al. 2018 Allicock et al. This content is distributed under the terms of the Creative Commons Attribution 4.0 International license.

### Probe design.

Our objective was to target all known human bacterial pathogens as well as any known antimicrobial resistance genes and virulence factors. Known human-pathogenic bacteria were selected from the available bacterial genomes in the PATRIC database (11). Included were all species for which at least one strain or isolate is annotated as “human-related” and “pathogenic.” One genome was selected per species due to probe number limitations. In consultation with experts in the field, we added other bacterial species that were considered to have high potential to become pathogenic. The final list contained 307 species (Table S1), including all 19 bacterial species listed in the priority list from the Child Health and Mortality Prevention program of the Bill and Melinda Gates Foundation.

The protein coding sequences from the selected genomes of the 307 species were extracted and combined with the full data set of 2,169 antimicrobial resistance gene sequences in the CARD database (12) and the 30,178 virulence factor genes in the VFDB database (13, 14). All databases were downloaded in March 2017. The combined target sequence data set (1,196,156 genes) was clustered at 96% sequence identity (resulting in 1,007,426 genes) and sent to the bioinformatics core of Roche Sequencing Solutions (Madison, WI), where sequences were subjected to further filtration based on printing considerations. Probe lengths were refined by adjusting their start/stop positions to constrain the melting temperature. The final library comprised 4,220,566 oligonucleotides with an average length of 75 nt. The average interprobe distance between probes along the targeted bacterial proteome, virulence, and AMR targets was 121 nt.

### Unbiased high-throughput sequencing.

Double-stranded DNA (for detection of bacteria) or cDNA (for detection of transcripts) was sheared to an average fragment size of 200 bp (E210 focused ultrasonicator; Covaris, Woburn, MA). Sheared products were purified using AxyPrep Mag PCR cleanup beads (Axygen/Corning, Corning, NY), and libraries were constructed using KAPA library preparation kits (Wilmington, MA) with input quantities of 10 to 100 ng DNA. Libraries were purified (AxyPrep), quantitated by Bioanalyzer (Agilent), and then split, with one half processed by BacCapSeq (see below) and the other directly sequenced on an Illumina MiSeq platform v3, with 150 cycles (San Diego, CA).

### Bacterial capture sequencing.

Nucleic acid preparation, shearing, and library construction were the same as for UHTS, except for the use of SeqCap EZ indexed adapter kits (Roche Sequencing Solutions, Pleasanton, CA) (BacCapSeq). The quality and quantity of libraries were checked using a Bioanalyzer (Agilent). Libraries were mixed with a SeqCap HE universal oligonucleotide, SeqCap HE index blocking oligonucleotides, and COT DNA and vacuum evaporated at 60°C. Dried samples were mixed with hybridization buffer and hybridization component A (Roche) prior to denaturation at 95°C for 10 min. The BacCapSeq probe library (SeqCap EZ Designs, v4.0; Roche) was added and hybridized at 47°C for 12 h in a standard PCR thermocycler. SeqCap Pure capture beads (Roche) were washed twice, mixed with the hybridization mixture, and kept at 47°C for 45 min with vortexing for 10 s every 10 to 15 min. The streptavidin capture beads complexed with biotinylated BacCapSeq probes were trapped (DynaMag-2 magnet; Thermo Fisher) and washed once at 47°C and then twice more at room temperature with wash buffers of increasing stringency. Finally, beads were suspended in 50 µl water and directly subjected to posthybridization PCR (SeqCap EZ accessory kit V2; Roche). The PCR products were purified (Agencourt Ampure DNA purification beads; Beckman Coulter, Brea, CA) prior to sequencing on an Illumina MiSeq platform v3. The time required for extraction, library construction, hybridization, generation of 150 bp single reads, and bioinformatic analysis is approximately 70 h.

### Data analysis and bioinformatics pipeline.

Each sample yielded an average of 5 million 100 bp single-end reads. The demultiplexed FastQ files were adapter trimmed using Cutadapt v1.13 (29). Adapter trimming was followed by generation of quality reports using FastQC v0.11.5 (https://www.bioinformatics.babraham.ac.uk/projects/fastqc/) and filtering with PRINSEQ v 0.20.3 (30). Host background levels were determined by mapping the filtered reads against the human genome using Bowtie2 v2.0.6 (31). The host-subtracted reads were *de novo* assembled using Megahit v1.0.4-beta (32); contigs and unique singletons were subjected to homology search using MegaBlast against the GenBank nucleotide database (33). The genomes of the tested bacteria were mapped with Bowtie2 using the filtered data set to visualize the depth and the genome recovery in IGV (34, 35). Results of spiking experiments are presented without any cutoff. Results of clinical blood culture and AMR treatment analyses are presented using an empirical cutoff. Targets with read counts above a 0.001% cutoff (>10 reads/1 million quality- and host-filtered reads) were rated positive.

For transcriptional analyses, MiSeq reads were aligned using the STAR read mapping package (36). Expression data were extracted from each sample using featureCounts (37), and the results were compiled into a master data file representing transcript counts for each gene. These data were normalized based on the number of reads sequenced for each sample, and the data were sorted by strain (AMR^+^/AMR^−^), time point, and antibiotic treatment to identify genes with differences in growth patterns based on these metrics.
